# Trends in conventional cardiovascular risk factors and myocardial infarction subtypes among young Chinese men with a first acute myocardial infarction

**DOI:** 10.1002/clc.23770

**Published:** 2021-12-28

**Authors:** Min Zhang, Hui‐Juan Zuo, Hong‐Xia Yang, Nan Nan, Xian‐Tao Song

**Affiliations:** ^1^ Department of Cardiology, Beijing Anzhen Hospital Capital Medical University Beijing China; ^2^ Department of Community Health Research Beijing Institute of Heart Lung and Blood Vessel Diseases, Beijing Anzhen Hospital, Capital Medical University Beijing China

**Keywords:** acute myocardial infarction, risk factor, trends, youth

## Abstract

**Background:**

There is limited data on the characteristics of conventional risk factors (RFs) in young Chinese men hospitalized with a first acute myocardial infarction (AMI).

**Hypothesis:**

We analyzed the trends in and prevalence of cardiovascular RFs and subtypes of MI during the first AMI in young Chinese men.

**Methods:**

A total of 2739 men aged 18–44 years hospitalized for a first AMI were identified from 2007 to 2017. The overall prevalence of RFs and their respective temporal trends and subtypes of AMI were evaluated.

**Results:**

The most prevalent conditions were smoking, followed by hypertension and then obesity. Patients aged <35 years had a much higher prevalence of hypercholesterolemia and obesity. Compared with a similar reference population in the United States, young Chinese men had a higher prevalence of smoking and dyslipidemia, but a lower prevalence of obesity, hypertension, and diabetes. The prevalence of hypertension increased from 2007 through 2017 (*p* trend <.001), whereas smoking decreased gradually. AMI frequently presented as ST‐segment elevation MI (STEMI) (77.5%). Cluster of conventional RFs (3 RFs, odds ratio [OR]: 1.69, 95% confidence interval [CI]: 1.11–2.57; ≥4 RFs, OR: 2.50, 95% CI: 1.55–4.03] and multivessel disease (OR = 1.32, 95% CI: 1.08–1.60) increased the risk of non‐STEMI (NSTEMI).

**Conclusions:**

Conventional RFs were highly prevalent in young Chinese men who were hospitalized for first AMI events, and the temporal trends varied different between China and US populations. Multivessel disease and cluster of conventional RFs are closely related to NSTEMI. Optimized preventive strategies among young adults are warranted.

## 1 INTRODUCTION

The primary and secondary prevention of coronary heart disease (CHD) in young adults has garnered tremendous attention given the rapid increase in the incidence of acute coronary events and hospitalization rates, especially in young men. Evidence from observational epidemiological studies showed the incidence of acute coronary events increased by 37.4% in the year 2009 compared to 2007 in young adults aged 35–39 years, making it the largest increase for this age group.^1^ Hospitalization rates for acute myocardial infarction (AMI) per 100 000 population experienced the most significant increase in young men (<55 years), by 45.8% from 2007 to 2012 in Beijing;^2^ the proportion of young adults hospitalized for CHD was nearly 90% in men from 2013 to 2014 in Beijing.^3^ This trend parallels an increase in cardiovascular risk factors (RFs) including smoking, hypertension, diabetes, obesity, and dyslipidemia in the general Chinese population as well as an increase in hospitalizations for AMI.^2^, ^4^, ^5^, ^6^

The Prospective Urban Rural Epidemiology (PURE) study showed that approximately 70% of cardiovascular disease (CVD) cases were attributed to modifiable RFs.^7^ Though several studies have evaluated the prevalence of these RFs during a first or any episode of AMI and have found a high prevalence of at least 1 RF (approximately 90%),^8^, ^9^, ^10^ most patients were classified as being at low or intermediate risk by traditional CHD risk prediction scores.^11^, ^12^ Unfortunately, this does not aid in the development of appropriate primary preventive strategies to decrease the risk of CHD. The prevalence of conventional RFs, other clinical characteristics, and their trends need to be clarified, which can be used in formulating preventive strategies.

Few studies have evaluated recent long‐term trends and prevalence of modifiable RFs during a first AMI in young adults in China. In a retrospective analysis of patients with coronary artery disease aged ≤45 years conducted from 2010 to 2014,^6^ the prevalence figures varied for the United States and German populations.^8^, ^10^ Contemporary data about trends in and prevalence of modifiable RFs, and subtypes of AMI are lacking in young patients. Using a retrospective analysis of hospital data from 2007 to 2017, we aimed to evaluate the trends in and prevalence of modifiable RFs and subtypes of MI during the first AMI in young Chinese men. This evidence will provide a reference point for the development of preventive strategies in this population.

## 2 METHODS

### 2.1 Participants

This study was based on a retrospective, single‐center analysis of young men hospitalized for a first AMI. Clinical and demographic data were collected from Beijing Anzhen Hospital by trained abstractors, using physician notes, laboratory reports, patient histories, and discharge summaries from January 2007 through December 2017. Young men aged 18–44 years hospitalized for a first AMI were identified from 2007 to 2017; 136 patients were sampled at the end of 2007 and a total of 2739 patients were recruited at the end of 2017 (Figure S1). The reference population for this analysis has previously been reported by Yandrapalli et al. Briefly, hospitalizations for a first AMI in young adults aged 18–44 years were identified from the US Healthcare Cost and Utilization Project (HCUP) National Inpatient Sample (NIS) from January 2005 through September 2015.^10^ Hospitalizations for AMI were determined according to the fourth universal definition of MI.^13^ AMI cases were first identified by excluding cases with secondary diagnoses of prior MI, prior percutaneous coronary intervention, prior coronary artery bypass grafting, post‐AMI syndrome, chronic ischemic heart disease, heart transplant recipient, and coronary arterial disease of bypass grafts or in transplanted hearts. Cases with a history of heart failure (HF), arteritis, congenital heart disease, and cancer were excluded. Those without coronary angiography and those with missing values for laboratory reports were excluded.

### 2.2 Measurements and diagnostic criteria

Primary outcomes of interest were the overall prevalence of the RFs and their respective temporal trends and subtypes of AMI. AMI was defined based on established criteria, which included^13^: detection of a rise and/or fall of cardiac biomarker values (preferably cardiac troponin [cTn]) with at least one value above the 99th percentile upper reference limit (URL) and with at least one of the following: (1) ischemia; (2) new or presumed new significant ST‐segment–T wave (ST–T) changes or new left bundle branch block; (3) development of pathological Q waves in the ECG; (4) imaging evidence of new loss of viable myocardium or new regional wall motion abnormality; and (5) detection of an intracoronary thrombus by angiography or autopsy. AMI was classified as ST‐segment elevation myocardial infarction (STEMI) and non‐STEMI (NSTEMI). Prevalent hypertension and diabetes were defined based on a documented history of hypertension and diabetes, respectively, in medical records. Hypercholesterolemia was defined as total cholesterol (TC) ≥ 5.2 mmol/L (200 mg/dl) or low‐density lipoprotein cholesterol (LDL‐C) ≥ 3.4 mmol/L (130 mg/dl). Obesity was defined as a body mass index (BMI) ≥ 28 (kg/m^2^). Smokers were defined as those who reported smoking cigarettes for >6 months. When the conventional RFs were compared with that of the US population, dyslipidemia and obesity were defined according to criteria in the report by Yandrapalli' et al.^10^

### 2.3 Statistical analysis

Categorical variables were expressed as total numbers (proportions), differences in RF prevalence across age groups were compared using *χ* ^2^ tests for categorical variables and trends in the prevalence of RFs were analyzed using linear‐by‐linear association. Normally distributed continuous variables were presented as means ± standard deviations. A student's *t* test was used to compare two independent samples for normally distributed continuous variables and a Mann–Whitney *U* test for continuous variables with skewed distributions. Odds ratios (ORs) with 95% confidence intervals (CIs) for associations were derived from multivariable logistic regression. All reported *p* values were two sided. Statistical analysis was performed using IBM SPSS Statistics version 25.0 (IBM).

## 3 RESULTS

### 3.1 Prevalence of conventional cardiovascular RFs and MI subtypes across different groups

In this study, 2739 cases of first AMI hospitalizations in young men were enrolled from January 1, 2007, to December 31, 2017. Of the total, 462 (16.9%) patients were <35 years, 761 (27.8%) were 35–39 years and 1516 (55.3%) were 40–44 years. The most frequent presentation of AMI was STEMI (77.5%) and over 80% underwent revascularization. Baseline characteristics of the overall sample and by age subgroups are presented in Table 1. The most prevalent RFs were smoking (75.5%), followed by hypertension (40.6%) and then obesity (38.3%). The proportion of patients without any of the five conventional CVD RFs was only 5.8% and 71.7% of patients had at least 2 RFs. Significant differences were noted for the prevalence of obesity, hypertension, diabetes, and hypercholesterolemia across age groups. Patients aged <35 years had a higher prevalence of hypercholesterolemia and obesity, with lower values for hypertension; the proportion of patients with 2 or 3 RFs in this age group was also higher than that for the other age groups.

**Table 1:** Baseline characteristics of the study population

Characteristics	Overall	<35 years	35–39 years	40–44 years	*p* value
	*n* = 2739	*n* = 462	*n* = 761	*n* = 1516	
AMI subtype					.372
STEMI	2123 (77.5)	349 (75.5)	601 (79.0)	1173 (77.4)	
NSTEMI	616 (22.5)	113 (24.5)	160 (21.0)	343 (22.6)	
Revascularization	2226 (81.2)	322 (69.7)	646 (84.9)	1258 (83.0)	<.001
Risk factors					
Obesity	1049 (38.3)	205 (44.4)	309 (40.6)	535 (35.3)	.001
Hypertension	1113 (40.6)	132 (28.6)	299 (39.3)	682 (45.0)	<.001
Diabetes	407 (14.9)	41 (8.9)	105 (13.8)	261 (17.2)	<.001
Hypercholesterol	726 (26.5)	162 (35.1)	198 (26.0)	366 (24.1)	<.001
Smoking	2068 (75.5)	349 (75.5)	592 (77.8)	1127 (74.3)	
Number of risk factors					.270
0	179 (6.5)	34 (7.4)	38 (5.0)	107 (7.1)	
≥1	2560 (93.5)	428 (92.6)	723 (95.0)	1409 (92.9)	
≥2	1748 (63.8)	301 (65.2)	484 (63.6)	963 (63.5)	
≥3	828 (30.2)	129 (28.0)	232 (30.4)	467 (30.8)	
≥4	207 (7.5)	27 (5.9)	59 (7.7)	121 (8.0)	

*Note*: Data are presented as mean ± standard deviation or *n* (%).

Abbreviations: AMI, acute myocardial infarction; NSTEMI, non‐ST‐segment elevation myocardial infarction; STEMI, ST‐segment elevation myocardial infarction.

### 3.2 Prevalence of conventional CVD RFs compared with a reference population

We compared the prevalence of classic modifiable cardiovascular RFs in the study population with that of the reference population. Compared with the reference population, young Chinese men had a higher prevalence of smoking (75.5% vs. 58.1%; rate difference 17.4%) and dyslipidemia (65.0% vs. 54.6%; rate difference 10.4%), with lower prevalence of obesity (13.1% vs. 18.6%; rate difference 19.7%), hypertension (40.6% vs. 49.3%; rate difference 8.7%), and diabetes (14.9% vs. 19.9%; rate difference 5.0%). The three leading conventional RFs in the reference population were smoking, dyslipidemia, and hypertension, and this was similar for the target population (Table 2).

**Table 2:** Prevalence of conventional cardiovascular risk factors in the study population compared with the reference population

Description	Target population	Reference population	*p* value
Age	39 ± 5	39 ± 5	－
Smoking	2 068 (75.5)	118 350 (58.1)	<.001
BMI ≥ 30	359 (13.1)	37 888 (18.6)	<.001
Diabetes	407 (14.9)	40 536 (19.9)	<.001
Dyslipidemia	1 780 (65.0)	111 220 (54.6)	<.001
Hypertension	1 113 (40.6)	100 421 (49.3)	<.001

*Note*: Data are presented as mean ± standard deviation or *n* (%).

Abbreviation: BMI, body mass index.

### 3.3 Conventional cardiovascular RFs and other clinical characteristics across AMI subtypes

Compared to STEMI patients, NSTEMI patients had a high prevalence of all individual conventional RFs except for smoking, which was similar in prevalence. The proportion of patients who had at least 3 RFs was 39.6% in NSTEMI patients, which was higher than that in STEMI patients (27.5%). The coronary artery involved most frequently was left anterior descending coronary artery (LAD) in both groups, but this was higher in STEMI patients. Patients with multivessel coronary disease and those without significant coronary stenosis were higher in the NSTEMI group (Table 3).

**Table 3:** Conventional risk factors and clinic characteristics according to AMI subtype

Description	STEMI patients	NSTEMI patients	*p* value
	*n* = 2123	*n *= 616	
Conventional risk factors			
Obesity	759 (35.8)	290 (47.1)	<.001
Hypertension	819 (38.6)	294 (47.7)	<.001
Diabetes	298 (14.0)	109 (17.7)	.025
Hypercholesterolemia	531 (25.0)	195 (31.7)	.001
Smoking	1595 (75.1)	473 (76.8)	.400
Number of conventional RFs			<.001
0	144 (6.8)	35 (5.7)	
1	684 (32.2)	128 (20.8)	
2	711 (33.5)	209 (33.9)	
3	451 (21.2)	170 (27.6)	
≥4	133 (6.3)	74 (12.0)	
Culprit coronary artery		177 (28.7)	
LMT	53 (2.5)	13 (2.1)	.065
LAD	1436 (67.6)	362 (58.8)	<.001
LCX	737 (34.7)	289 (46.9)	<.001
RCA	963 (45.4)	261 (42.2)	.169
Number of vessels involved			<.001
Without significant coronary stenosis or normal	175 (8.2)	73 (11.9)	
Single vessel disease	992 (46.7)	226 (37.6)	
Multivessel coronary disease	956 (45.0)	317 (51.5)	

*Note*: Data are presented as *n* (%).

Abbreviations: EF, ejection fraction; LAD, left anterior descending coronary artery; LCX, left circumflex coronary artery; NSTEMI, non‐ST elevation acute coronary syndrome; RCA, right coronary artery; STEMI, ST‐segment elevation myocardial infarction.

We also conducted logistic regression analyses to evaluate the relationships between AMI subtype and conventional RFs. Multiple conventional RFs significantly increased the risk of NSTEMI; the OR was 1.69 (95% confident interval [CI]: 1.11–2.57) for patients with three RFs and 2.50 (95% CI: 1.55–4.03) for patients with at least 4 RFs. Patients with multivessel coronary disease (OR = 1.32, 95% CI: 1.08–1.60) and patients without significant coronary stenosis or normal patients (OR = 2.01, 95% CI: 1.47–2.76) had an increased risk of NSTEMI compared with those with the single vessel disease after adjusting for the other covariates (Table 4).

**Table 4:** Relationship between AMI subtype and conventional RFs and coronary artery disease

Factors	Univariate analysis		Muitivariate analysis	
	OR (95% CI)	*p* value	OR (95% CI)	*p* value
Age group	0.97 (0.87–1.10)	.671	0.97 (0.86–1.09)	0.006
Family history of CHD	0.811 (0.62–1.06)	.128	0.76 (0.58–1.04)	0.053
Number of conventional RFs				
0	1		1	
1	0.77 (0.51–1.17)	.217	0.82 (0.54–1.25)	0.355
2	1.21 (0.81–1.81)	.352	1.31 (0.87–1.97)	0.194
3	1.55 (1.03–2.34)	.036	1.69 (1.11–2.57)	0.014
≥4	2.29 (1.44–3.65)	<.001	2.50 (1.55–4.03)	<0.001
Number of vessels involved				
Single vessel disease	1		1	
Multivessel coronary disease	1.45 (1.20–1.76)	<.001	1.32 (1.08–1.60)	0.007
Without significant coronary stenosis or normal	1.93 (1.34–2.49)	<.001	2.01 (1.47‐2.76)	<0.001

### 3.4 Trends of conventional RFs and AMI subtypes

**Figure 1:**
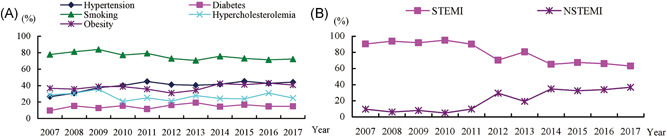
Trends in the percentage of five conventional risk factors and acute myocardial infarction (AMI) subtype during a first acute myocardial infarction in young men 18–44 years old between 2007 and 2017. (A) Trends in the percentage of conventional risk factors, the increasing prevalence was noted for hypertension (*p* trend = 0.004). Decline in the rates of current smoking and drinking are appreciated. There was a similar prevalence for diabetes and hypercholesterolemia and obesity over time. (B) Trends in the percentage of AMI subtybe, the Increasing percentage was noted for NSTEMI(*p* trend <0.001)

The trends in the prevalence of conventional RFs are shown in Figure 1A. Compared with 2007, the prevalence of hypertension increased in 2017 (*p* trend = 0.004) that for smoking decreased gradually (*p* trend <0.05) and those for diabetes, hypercholesterolemia, and obesity remained unchanged (*p* trend >0.05). Between 2007 and 2017, the prevalence of hypertension increased from 26.5% to 44.2% (rate difference 17.7%) and that for smoking decreased from 77.9% to 72.4% (rate difference 5.5%). The prevalence of hypertension increased through 2007 and 2011, and then maintained a little shift. The greatest relative increase in prevalence between 2007 and 2009 was observed for hypercholesterolemia (from 28.7% to 35.5%), decreased in 2010 (from 35.5% to 20.5%), and then maintained a little shift. There were little shifts for diabetes and obesity (diabetes, 9.6% in 2007 and 14.8% in 2017, *p* = .283, rate difference 5.2%; obesity, 36.8% in 2007, and 40.7% in 2017, *p* = .105, rate difference 3.9%).

The most frequent presentation of AMI was STEMI, but the proportion decreased gradually from 90.4% to 63.2% (*p* trend <0.001), then stabilized at around 65% during the current years (Figure 1B).

The mean SBP and BMI increased over the three periods; the mean SBP increased from 119.4 mmHg to 123.3 mmHg (mean difference 3.9 mmHg), with BMI increasing from 26.5 kg/m^2^ to 27.6 kg/m^2^ (mean difference 1.2 kg/m^2^). The mean TC decreased from 4.62 mmol/L to 4.49 mmol/L and LDL‐C decreased from 2.97 mmol/L to 2.80 mmol/L. No differences were seen in mean DBP and FPG over the three periods (Table S1).

## 4 CONTROL OF CONVENTIONAL RFS

The control rate of hypertension and diabetes was 62.8% (699/1113) and 17.7% (72/407), respectively; smoking cessation was 4.8% (105/2173).

## 5 DISCUSSION

In this study comprising of young men with a first hospitalization for AMI in China, the most prevalent CVD RFs were smoking (75.5%), hypertension (40.6%), and obesity (38.3%). Additionally, over 70% of them had at least two conventional RFs. Compared with a similar reference population, these young Chinese men had a higher prevalence of smoking and dyslipidemia and a lower prevalence of obesity, hypertension, and diabetes. Between 2007 and 2017, the prevalence of hypertension increased, whereas that of smoking decreased. Patients presenting with NSTEMI had a high prevalence of all individual conventional RFs except for smoking, which was more prevalent in STEMI patients. LAD was the most frequently involved coronary artery in the subtypes of AMI, but it was more common in STEMI patients. Multivessel coronary disease was more common in NSTEMI.

Conventional RFs were highly prevalent in young men aged 18–44 years who were hospitalized for first AMI events. The prevalence values were higher compared to national figures smoking (53.9% for young adults aged 25–44 years), hypertension (11.3% for urban adults and 10.0% for rural adults aged 18–44 years), obesity (11.0% for young adults aged 18–44 years), and diabetes (5.9% for young adults aged 18–44 years); high TC ( ≥ 5.2 mmol/L) and high LDL‐C( ≥ 3.4 mmol/L) were 28.5% and 26.3%, respectively.^14^, ^15^, ^16^, ^17^ Our results are consistent with previous studies conducted in young patients diagnosed with AMI and CHD; the prevalence for hypertension, diabetes, and smoking has been reported as 34.3%–41.7%, 11.1%–22.4%, and 57.4%–74.0%, respectively.^3^, ^6^ However, the prevalence figures were different from that of the US report. Compared with the data from the US NIS for patients hospitalized for a first AMI from 2005 to 2015,^10^ young Chinese men had a higher prevalence of smoking and dyslipidemia, with a lower prevalence of hypertension, obesity, and diabetes. There were clear age differences in the prevalence of some of the RFs; patients aged <35 years had a higher prevalence of smoking, obesity, and hypercholesterolemia, whereas patients aged 35–44 years had a higher prevalence of smoking, hypertension, and diabetes.

Our study also showed differences in the temporal trends of the conventional RFs between China and US populations. The prevalence of all the evaluated conventional RFs progressively increased between 2005 and 2015 in the United States,^10^ but hypertension prevalence increased in China, whereas smoking decreased gradually; which was also consistent with a previous study conducted in China. In a retrospective analysis of coronary artery disease patients aged ≤45 years conducted from 2010 to 2014, the prevalence of hypertension increased from 40.7% to 47.5%, 20.3% to 26.1% for diabetes, and 27.3% to 35.7% for hyperlipidemia. However, the prevalence of smoking exhibited a downward trend.^6^

Approximately 77% of young Chinese men hospitalized with their first MI presented with STEMI, which was consistent with other studies conducted in young Chinese adults.^2^, ^18^, ^19^ Previous population‐based studies have, however, reported NSTEMI to be the most common presentation^2^, ^19^; the proportion of patients presenting with NSTEMI increased from 11.6% to 36.2% in males and from 15.8% to 45.5% in females from 2007 through 2012,^2^ which was quite different from the presentation in young adults in the US population.^10^, ^20^, ^21^ Latest studies conducted in the United States have shown that the presentation of AMI was more frequently NSTEMI and increased from 54.6% to 67.9% in males and from 58.8% to 80.2% in females from 2000 through 2014.^21^ A similar trend has been observed in Germany.^8^ According to our findings, multivessel disease and cluster of conventional RFs are closely related to NSTEMI, which are consistent with the results of previous studies.^10^, ^22^

In our study, we focused on young adults at high risk of CVD in routine clinical practice; but most of these young adults are classified as low or intermediate risk based on traditional CVD risk prediction scores that use lifetime risk estimates.^11^, ^12^ Using this approach leads to decreased ability to identify RFs in young adults and suboptimal utilization of preventive strategies. Furthermore, the majority of CHD events seem to occur in these “low” and “intermediate” risk groups. The knowledge of the prevalence and trends of conventional RFs among young Chinese men hospitalized with first MI have important healthcare implications with regard to planning appropriate primary preventative strategies. Considerable attention should be paid to young men with at least two out of five conventional RFs (hypercholesterolemia, hypertension, diabetes, obesity, smoking). Healthcare providers should also focus more attention on the control of metabolic factors and encourage smoking cessation.

## 6 LIMITATIONS

There are some limitations that deserve consideration. This was a single‐center study and the data were collected from Beijing Anzhen hospital, which is well known for the management of CHD. Furthermore, some patients may have more severe symptoms. Hence, the results may not be extrapolated to the entire nation. Data on conventional RFs were collected from medical records; therefore, health behaviors such as physical activity, sleep duration, emotion, and stress could not be included. The sample for the annual number of AMI hospitalizations was not large enough, which may have influenced the comparisons of conventional RFs and trends in RFs over time with the reference US population.

## 7 CONCLUSIONS

In conclusion, the characteristics of conventional RFs among young Chinese men hospitalized with a first MI have important healthcare implications with regard to the planning of appropriate primary preventative strategies. The three leading cardiovascular RFs (smoking, hypertension, and obesity) should be the target of intervention and treatment strategies aimed at reducing the incidence of MI in young adults. Furthermore, preventing hypercholesterolemia in patients aged 34 years or younger. should also be a primary focus. Young adults with two or more conventional RFs should be given considerable attention even if they were classified as being at low or intermediate risk by traditional CHD risk prediction scores.

## CONFLICT OF INTERESTS

The authors declare that there are no conflict of interests.

8

## Supporting information

Supplementary information.

Supplementary information.

## Data Availability

The data that support the findings of this study are available from the corresponding author upon reasonable request.
