# Supplementary figures and images for: Trends in conventional cardiovascular risk factors and myocardial infarction subtypes among young Chinese men with a first acute myocardial infarction
Source: Clin Cardiol. 2021 Dec 28;45(1):129–35. doi: 10.1002/clc.23770 (PMC8799041; doi:10.1002/clc.23770)

(Number)

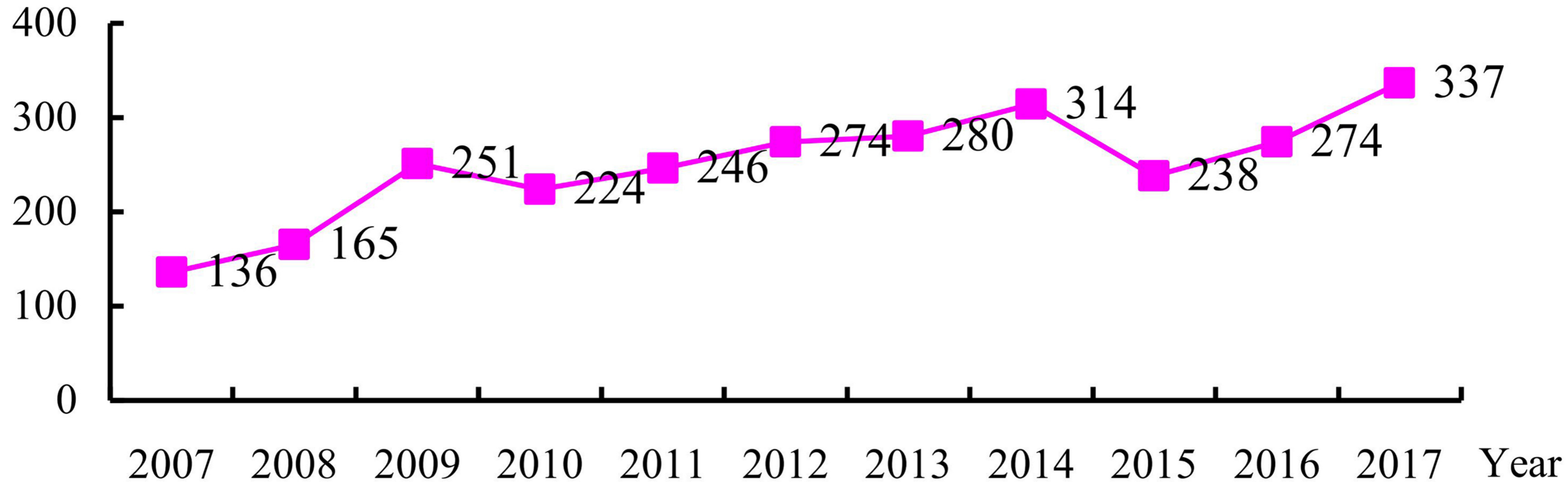

Supplement: Supplementary file 1 — Supplementary information. [file CLC-45-129-s001.pdf]
