# Supplementary material for: Trends in conventional cardiovascular risk factors and myocardial infarction subtypes among young Chinese men with a first acute myocardial infarction
Source: Clin Cardiol. 2021 Dec 28;45(1):129–35. doi: 10.1002/clc.23770 (PMC8799041; doi:10.1002/clc.23770)
Supplement: Supplementary file 2 — Supplementary information. [file CLC-45-129-s002.docx]

**Supplementary Table**  Mean levels of conventional RFs across the three periods

| Conventional RFs level | Overall  (n=2739) | 2007-2009  (n=552) | 2010-2013  (n=1024) | 2014-2017  (n=1163) | P-value |
| --- | --- | --- | --- | --- | --- |
| SBP (mmHg) | 121.5±16.8 | 119.4±17.9 | 120.7±16.6 | 123.3±16.2 | <0.001 |
| DBP (mmHg) | 76.8±12.1 | 76.9±12.5 | 76.7±11.9 | 76.8±12.1 | 0.931 |
| FPG (mmol/L) | 7.18±4.27 | 6.97±2.83 | 7.42±5.13 | 7.07±4.01 | 0.107 |
| TC (mmol/L) | 4.49±1.20 | 4.62±1.34 | 4.41±1.14 | 4.49±1.17 | 0.006 |
| LDL-C (mmol/L) | 2.83±0.96 | 2.97±1.03 | 2.77±0.92 | 2.80±0.96 | <0.001 |
| BMI (kg/m^2^) | 27.2±3.7 | 26.5±4.1 | 26.9±3.4 | 27.6±3.8 | <0.001 |
